# Association between weekly fruit and vegetable consumption and depressive symptoms: results from the Korean Elderly Environmental Panel study

**DOI:** 10.4178/epih.e2021029

**Published:** 2021-04-20

**Authors:** Youjeong Yuk, Chae-Rin Han, Yoonyoung Jang, Yun-Chul Hong, Yoon-Jung Choi

**Affiliations:** 1 Seoul National University College of Medicine, Seoul, Korea; 2 Department of Preventive Medicine, Seoul National University College of Medicine, Seoul, Korea; 3 Environmental Health Center, Seoul National University College of Medicine, Seoul, Korea; 4 Institute of Environmental Medicine, Seoul National University Medical Research Center, Seoul, Korea

**Keywords:** Depression, Fruit, Vegetables, Aged, Korean, Korean version of Short form Geriatric Depression Scale

## Abstract

**OBJECTIVES:**

Although previous studies have investigated the correlation between fruit and vegetable consumption and depression, the results remain inconclusive. The present study aimed to investigate the association between weekly fruit and vegetable consumption and depressive symptoms in elderly Koreans.

**METHODS:**

A multiple covariate linear regression analysis was performed using the data of 1,226 elderly individuals ≥ 60 years of age who participated in the Korean Elderly Environmental Panel II study from 2012 to 2014. Depressive symptoms were evaluated using the Korean version of the Short form Geriatric Depression Scale (SGDS-K). Generalized linear mixed-effects models were constructed to analyze the repeated measurements of 305 people who participated in the survey every year.

**RESULTS:**

After adjusting for confounders, SGDS-K scores were negatively associated with the frequency of weekly fruit consumption as follows (β [95% confidence interval; CI]: -0.17 [-0.28 to -0.05], -0.17 [-0.27 to -0.07], -0.42 [-0.54 to 0.29], and -0.33 [-0.44 to -0.21]) for less than 1 time/wk, 1-3 times/wk, 4-6 times/wk, and daily, respectively, compared to no consumption. The SGDS-K scores were also negatively associated with the frequency of vegetable consumption (β [95% CI]: -0.86 [-1.18 to -0.55], -0.18 [-0.35 to -0.01], -0.36 [-0.53 to -0.18], and -0.15 [-0.29 to 0.00]) in the above order, respectively.

**CONCLUSIONS:**

Fruit consumption was inversely associated with depression levels in a dose-dependent manner. Although there was no dose-response relationship between vegetable consumption and the level of depression, it was negatively associated with SGDS-K scores.

## INTRODUCTION

Depression is a major public health problem with an increasing incidence and resultant global burden [1]. Other chronic diseases can exacerbate the symptoms of depression [2]. Hence, it is important to identify preventive or curative methods to prevent or relieve symptoms in patients with depression. Several previous studies have investigated the relationship between fruit and vegetable consumption and depressive symptoms. It is postulated that fruit and vegetable consumption may alleviate depressive symptoms, as they contain antioxidants and minerals that are involved in the synthesis of neurotransmitters. Nevertheless, the results are inconsistent [3-11], which could be attributed to variations in dietary patterns among different countries. Consequently, each country should conduct individual studies to evaluate the effects of fruit and vegetable consumption on depression.

Korea has a high prevalence of depression and suicide. However, very few studies have investigated the association between fruit and vegetable consumption and depressive symptoms among elderly Koreans [12-15]. Among the Organization for Economic Cooperation and Development countries, Korea recorded the highest suicide rate among adults above the age of 70 years [16]. The incidence of depression increases exponentially with age [17]. In Korea, the highest suicide rate is observed among individuals above the age of 80 years, followed by individuals above the age of 70 years [16]. Elderly individuals have restrictions regarding physical and social activities and are at risk of consuming a nutritionally inadequate diet [17,18]. Hence, research should focus on the dietary behavior of the elderly, which can be easily improved. However, to the best of our knowledge, no previous study has focused solely on Korean elderly people regarding this particular aspect. The current study aimed to investigate the associations between weekly fruit and vegetable consumption and depressive symptoms in elderly Korean individuals.

## MATERIALS AND METHODS

### Participants and data source

**Figure 1.**
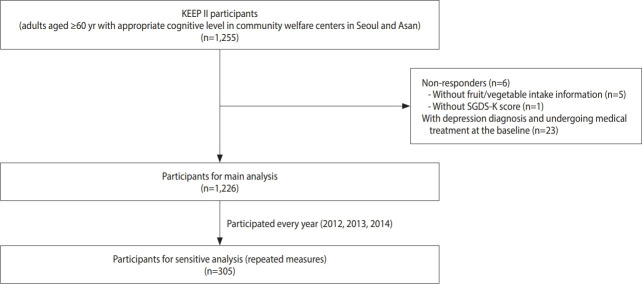
Selection of participants. KEEP, Korean Elderly Environmental Panel; SGDS-K, Korean version of the Short form Geriatric Depression Scale.

The Korean Elderly Environmental Panel (KEEP) study is a community-based, repeated-measures survey that was established to study the effects of diverse environmental exposures on the health of elderly Koreans [19]. The study methods and details are described in the literature [19]. Briefly, the KEEP study is composed of the KEEP-I and the KEEP-II. In KEEP-I, 560 elderly subjects were recruited in Seoul and followed up five times (six surveys) between 2008 and 2010, and in KEEP-II, 800 elderly subjects were recruited (400 from Seoul and 400 from Asan) and followed up twice (three surveys). Our study used data from the KEEP-II, which was conducted annually from 2012 to 2014. Most of the questionnaires, including weekly fruit/vegetable intake and the Korean version of the Short form Geriatric Depression Scale (SGDS-K), were investigated every year. The study participants were elderly adults aged ≥ 60 years with an appropriate cognitive level to understand the instructions of the survey, who regularly visited one of the two community welfare centers in the Seongbuk-gu area of Seoul and Asan, Korea. Of the 800 KEEP-II participants recruited in 2012, 416 and 327 participants were followed up in 2013 and 2014, respectively. New enrollment included 384 and 71 participants in 2013 and 2014, respectively. More details regarding follow-up status in Seoul and Asan are presented in Supplementary Material 1. From the baseline data of 1,255 individuals who participated in the study between 2012 and 2014, 5 participants whose response to the query regarding weekly fruit or vegetable consumption was “do not know,” 1 participant without SGDS-K information, and 23 participants who were diagnosed with depression and undergoing treatment at the baseline were excluded. However, subjects with depression who were not receiving therapy were included. Among the study subjects, 305 individuals who participated in the KEEP-II for three consecutive years were selected for the repeated-measures analysis (Figure 1).

### Assessment of depression

The current study used the SGDS-K as a screening tool to assess depression. The SGDS-K comprises 15 questions that evaluate the participant’s mood in the preceding week. The participants choose the response “yes” or “no,” with “yes” counted as 1 and “no” counted as 0 to yield a range of total score from 0 to 15. As the SGDS-K score increases, people experience more depressive symptoms. A score of 8 was used as the cut-off point for the diagnosis of depression. Previous studies have established that the SGDS-K has high validity for the diagnosis of depression [20].

### Dietary assessment

The dietary assessment was performed using a self-reporting method of responses to simple questions regarding fruit and vegetable consumption: “How often do you usually eat fruit? Fruits include fresh, dried, canned, and frozen fruit.” and “How often do you usually eat vegetables? Vegetables include fresh, dried, canned, and frozen vegetables.” The following choices were provided: (a) never, (b) less than 1 time/wk, (c) 1-3 times/wk, (d) 4-6 times/wk, (e) daily, and (f) do not know. The data signified the frequency, not the quantity, of consumption.

More detailed dietary information including the intake of each food item in grams, total energy intake, and daily nutrient intake were investigated for the year 2012 by using a semiquantitative food frequency questionnaire (FFQ) to assess the frequency of consumption (9 categories from rarely eaten to eaten more than 3 times/d) and portion size (categorized as small, average, or large) of 118 food items [21,22]. The amount of each food item was transformed into grams using the Computer Aided Nutritional Analysis Program for Professionals (CAN-pro 4.0, Korean Society of Nutrition, Seoul, Korea). However, because only 385 participants participated in the FFQ and the FFQ was conducted only in 2012, we decided to use the simple questions mentioned above.

### Other covariates

Age was categorized into three groups: 60-69 years, 70-79 years, and ≥ 80 years. Education level was categorized into three levels (detailed information provided in Supplementary Material 2). Allowance was categorized as < 30.00, 30.00-69.99, and ≥ 70.00 units of 10,000 Korean won (KRW) per month (approximately US$10). Only current behavior was considered to assess smoking status and alcohol consumption. Household type was categorized as living alone or living with family, which refers to living with spouse, sons or daughters, or others. The presence or absence of underlying diseases was assessed. Disease status refers to a history of admission/surgery/emergency room visit within 1 year or a visit to a doctor without admission within 2 weeks. We used the short form of the International Physical Activity Questionnaire to assess physical activity levels. Physical activity was coded as < 600 or ≥ 600 metabolic equivalents of task (MET) min/wk, with MET measured as energy expenditure in calories per kilogram of body weight for 1 minute (kcal/min/kg) (Supplementary Material 2). Body mass index (BMI) was calculated as weight divided by height squared and categorized as <23.0 kg/m^2^, 23.0-24.9 kg/m^2^, and ≥25.0 kg/m^2^, with ≥ 23.0 kg/m^2^ classified as overweight according to the Korean Society for the Study of Obesity [23]. According to the Korean Society for the Study of Obesity, ≥ 25.0 kg/m^2^ is classified as obese, although this value is classified as overweight by the World Health Organization [23,24].

### Statistical analysis

The t-test was used to compare continuous variables such as age and total energy intake in participants with depression (SGDS-K ≥ 8) and without depression (SGDS-K< 8), whereas chi-square tests were used to compare categorical variables such as sex, age category, education, allowance, smoking status, alcohol consumption status, household type, disease status, physical activity, BMI category, weekly fruit intake, and weekly vegetable intake.

The SGDS-K was evaluated as both a continuous and categorical variable. The SGDS-K score as a continuous variable refers to the level of depression, and as a categorical variable, it refers to depression status (SGDS-K ≥ 8 or < 8). The association between the level of depression and the frequency of fruit or vegetable consumption was also estimated. Multivariate linear regression was used to estimate β, 95% confidence interval (CI), and p-value for SGDS-K scores (continuous variable). Multivariate logistic regression was used to estimate odds ratios (ORs), 95% CIs, and p-values for depression status (SGDS≥ 8 or < 8). The analyses were performed using three models: a crude model, an age- and sex-adjusted model, and a multiple covariate-adjusted model. The confounding factors used in the multiple covariate-adjusted models were sex, age category (the age- and sex-adjusted model used age as a continuous variable), education, allowance, smoking status, alcohol consumption status, household type, disease status, physical activity, and BMI.

For sensitivity analysis, reciprocal adjustment for weekly vegetable intake and weekly fruit intake was performed in multiple covariate-adjusted models. Also, we performed sensitivity analysis by combining “never” and “less than 1 time” together as the sample sizes of these categories were small. Furthermore, generalized linear mixed-effects models were used for repeated measures in the subset of subjects who repeatedly participated in the KEEP survey 3 times. Linear mixed-effects models were also constructed as crude, age- and sex-adjusted, and multiple covariate-adjusted models.

To assess dose-response relationships, we regarded the frequency of weekly fruit and vegetable intake as a continuous variable; “never,” “less than 1 time/wk,” “1-3 times/wk,” “4-6 times/wk,” and “daily” were converted into 0 times/wk, 0.5 times/wk, 2 times/wk, 5 times/wk, and 7 times/wk, respectively, as these values are the average value of each category. We then assessed the dose-dependent relationship of the level of depression, that is, SGDS-K score (continuous variable), and depression status (SGDS-K < 8 or ≥ 8) with fruit and vegetable consumption using linear regression and logistic regression analyses, respectively. R version 4.0.3. (https://cran.r-project.org/) and SPSS version 25.0 (IBM Corp., Armonk, NY, USA) were used for the statistical analysis. In the present study, a p-value < 0.05 was considered statistically significant.

### Ethics statement

All participants provided written informed consent, and the study protocol was approved by the Institutional Review Board (IRB) of the Seoul National University College of Medicine (IRB No. 0804-045-241).

## RESULTS

The baseline characteristics of the participants are shown in Table 1. Among the 1,226 participants, 19.7% had depression. Compared to the participants without depression, the participants with depression were more likely to be female (78.0 vs. 67.1%) and older (75.53 vs. 73.94 years) and to have a lower educational level (less than middle school graduation: 77.2 vs. 65.5%), live alone (49.0 vs. 29.3%), and have an underlying disease (60.6 vs. 46.3%). Participants with depression were less likely to be physically active (< 600 MET min/wk: 70.7 vs. 60.5%), consume adequate calories (1,470.37 vs. 1,804.80 kcal/d), and consume fruits frequently (never: 19.9 vs. 9.6%; less than once/wk: 14.1 vs. 12.2%; 1-3 times/wk: 33.6 vs. 28.5%; 4-6 times/wk: 9.1 vs. 16.0%; and daily: 23.2 vs. 33.6%). However, the current study did not observe any significant differences between the groups with and without depression with regard to smoking status, alcohol consumption, BMI, and weekly vegetable intake (Table 1).

The association between weekly fruit intake, SGDS-K score, and depression status is shown in Table 2. The SGDS-K score was significantly and negatively associated with the frequency of fruit consumption in the crude, age- and sex-adjusted, and multiple covariate-adjusted models. The effect size of the association between fruit consumption and SGDS-K score increased with an increase in the frequency of fruit consumption. For instance, the β estimation for the association between fruit consumption frequency and SGDS-K score in the subjects who consumed fruits 1-3 times/wk was -0.17 (95% CI, -0.27 to -0.07), compared to the group with no fruit consumption. Fruit consumption at a frequency of 4-6 times/wk showed a greater association with SGDS-K scores (β, -0.42; 95% CI, -0.54 to -0.29). The OR of depression in the elderly subjects who consumed fruits 4-6 times/wk was 0.42 (95% CI, 0.23 to 0.75), compared to the group with no fruit consumption, whereas the OR pertaining to the group with daily fruit consumption was greater (OR, 0.55; 95% CI, 0.34 to 0.89) (Table 2).

The associations of weekly vegetable intake with the SGDS-K score and depression status are shown in Table 2. The SGDS-K scores were negatively associated with vegetable consumption frequency in the subjects who consumed vegetables 4-6 times/wk (β, -0.36; 95% CI, -0.53 to -0.18), compared to the subjects who did not consume vegetables. The association between vegetable consumption and depression status was not significant (Table 2).

### Sensitivity analysis

Reciprocal adjustment for fruit or vegetable consumption frequency did not affect the overall results (Supplementary Materials 3 and 4). Furthermore, as we grouped “never” and “less than once/ week” together (Supplementary Materials 5 and 6), we found that the trends of results were not different from the main analyses. The present study performed additional analyses using generalized linear mixed-effects models to analyze the repeated measures pertaining to the subjects who participated in the survey three consecutive times (n= 305). The risk of depression decreased with an increase in the weekly frequency of fruit consumption in a dosedependent manner. In the multiple covariate-adjusted model, the ORs for depression were 1.34 (95% CI, 0.48 to 3.71), 0.66 (95% CI, 0.27 to 1.60), 0.53 (95% CI, 0.18 to 1.56), and 0.40 (95% CI, 0.16 to 1.02) at weekly frequencies of less than 1 time/wk, 1-3 times/wk, 4-6 times/wk, and daily. However, significant results were observed only in relation to weekly fruit consumption of 4-6 times/wk in the crude model and the daily fruit consumption in the crude and age- and sex-adjusted models (Table 3). There was no significant association between vegetable consumption and the level of depression or depression status with regard to repeated measures (Table 3).

### Dose-dependent relationship

Regarding the dose-dependent relationship, the SGDS-K scores had a negative linear relationship with the frequency of weekly fruit consumption (β, -0.04; 95% CI, -0.06 to -0.03). This pattern was consistent in the linear mixed-effects model (β, -0.04; 95% CI, -0.06 to -0.03). The greater the frequency of fruit consumption, the lower the risk of depression in both the cross-sectional model (OR, 0.90; 95% CI, 0.84 to 0.97) and repeated measures (OR, 0.87; 95% CI, 0.78 to 0.96), when the frequency of fruit consumption was considered as a continuous variable. The linearity was not significant in the case of vegetable consumption (Table 4).

## DISCUSSION

The current study showed that fruit consumption was inversely associated with the level of depression in a dose-dependent manner. Although the level of depression did not have a dose-dependent relationship with vegetable consumption frequency, vegetable consumption significantly lowered the SGDS-K scores compared to no vegetable consumption.

Previous studies have reported inconsistent results regarding the association between the level of depression and fruit or vegetable consumption. Nevertheless, a couple of studies have reported that fruit consumption is more inversely associated with depressive symptoms than vegetable consumption, which is consistent with the current results [6,8]. However, certain studies have reported significant associations in relation to both fruit and vegetable consumption [10,11], whereas other studies did not detect such significant associations [7,9].

The frequency of vegetable consumption did not show a dosedependent relationship with the level of depressive symptoms and was not significantly associated with depression status. This finding could be attributed to the fact that more than 70% of the participants responded that they consumed vegetables every day, whereas less than 30% of the participants with depression responded with other choices. Moreover, the distribution of vegetable intake frequency in the subjects with and without depression was similar, which is mainly attributable to the fact that most Korean foods are vegetable-based and most Koreans consume vegetables every day. A basic Korean meal includes rice, soup, and kimchi, which are vegetable-based foods, in addition to soybean or vegetable-based dishes [25]. The availability of more comprehensive data is needed in future studies. For instance, certain cross-sectional studies have shown a dose-dependent relationship between the amount of fruit and vegetable intake and the level of depression [5,26].

### Plausible mechanisms

Fruits and vegetables contain several nutrients that are known to prevent depressive symptoms. Defects in antioxidant defense systems are associated with a high incidence of depression. This is because depression increases the production of proinflammatory cytokines, which induce reactive oxygen species, which then trigger the lipid peroxidation process, compromising the brain [27,28]. Hence, antioxidants such as vitamins C, E, and β-carotene play an important role [3,27]. For example, vitamin E prevents peroxidation of ω3 polyunsaturated fatty acid [25]. Nutrients such as folate are essential for the biosynthesis of neurotransmitters such as serotonin, which stabilizes mood, as folate acts as a methyl donor, which is essential for the conversion of homocysteine to methionine by methylation [29]. Thus, folate deficiency is associated with a high risk of depression as neurotransmitters such as serotonin decrease [30]. Vitamin B6 is also known to mitigate the symptoms of hormone-related depression, as vitamin B6 is also involved in the synthesis of neurotransmitters that regulate emotions [31].

Conversely, a plausible explanation for the inverse association between depression and fruit consumption, in contrast to vegetable consumption, is that fruits can substitute for sugar-rich desserts. As sugar consumption has been associated with a higher incidence of depression in six countries [32], eating fruits instead of a sugarrich dessert may have a more desirable effect in preventing depressive symptoms. Additionally, several antioxidative nutrients, such as tangeretin, nobiletin, rutin, and resveratrol, are present only in fruits and not in vegetables [31].

### Strengths

First, the data were analyzed for both the level and risk of depression. Most previous studies focused on the OR of depression, whereas the present study also presented the level of depressive symptoms using the SGDS-K scores. Second, unlike most crosssectional studies, the present study conducted a longitudinal analysis using a generalized linear mixed-effects model. Lastly, to the best of our knowledge, this is the first study to analyze the associations between the level of depressive symptoms, which is explained by the SGDS-K score, and fruit and vegetable consumption in elderly Korean people exclusively, although a few studies have involved Korean adults [12,13] and adolescents [14,15]. Ji et al. [12] included female subjects below 70 years of age, which may not represent the elderly Korean population. A study by Ju & Park [13] covered both the sex and age of elderly subjects; however, vegetable and fruit consumption were not analyzed separately.

### Limitations

First, only the frequency of dietary intake was included in the analyses. Hence, data regarding the quantity, quality, cooking methods, nutrient density, and the proportion of fruits and vegetables in the total dietary intake were unavailable. In addition, it was not clear if fruit juice or kimchi was included in the fruit or vegetable consumption, considering the character of the question in the questionnaire. However, as the current study involved elderly subjects and considering that the KEEP-II study included numerous questions that were answered repeatedly every year, enquiries regarding the usual dietary behavior, such as the weekly frequency of consumption, may be more realistic and intuitive for the participants than enquiries about the exact quantity of each food item, which results in higher data reliability.

Second, depression was not assessed using clinical diagnoses; instead, the SGDS-K was used as a screening tool. However, considering that the subjects were elderly, feeling depressed may not warrant a visit to the doctor, as they are reluctant to burden their families and do not perceive depression as a serious condition. Accordingly, SGDS-K may be more inclusive.

Third, this study used a cross-sectional analysis. Hence, the causality of fruit and vegetable consumption with respect to the level of depression could not be proven. As depression can alter dietary style, reverse causation cannot be excluded in the correlation between the variables. Further longitudinal or cohort studies are needed to confirm the causality.

Fourth, more frequent fruit or vegetable intake, such as twice or thrice a day, was not considered. For example, elderly Korean individuals often eat kimchi at every meal. Thus, for future studies, a clearer representation of the food category is required.

Lastly, because the survey used a self-reporting method, recall bias cannot be excluded, especially in older adults. Furthermore, there may be residual confounding factors, such as total energy intake, which was obtained in only a small number of people who participated in the FFQ in 2012 (n= 385); thus, BMI was used instead. Cognitive status was not considered, as the participants of the KEEP survey were required to have an adequate cognitive level. Therefore, the current study is only applicable to fully cognitive elderly individuals with typical Korean dietary behavior.

In conclusion, the present study implies that the level of depression, which is explained by the SGDS-K score, is inversely associated with the frequency of fruit intake in a dose-dependent manner. Vegetable consumption did not display a dose-dependent relationship with depression level. However, SGDS-K scores were significantly negatively associated with vegetable intake compared to no vegetable consumption. Further studies are required to elucidate the underlying mechanisms and to identify the causality between the frequency of fruit and vegetable intake and the level of depression.

## Figures and Tables

**Table 1.** General characteristics of study participants

Characteristics	No depression	Depression^1^	Total	p-value^2^
Total	985 (80.3)	241 (19.7)	1,226 (100)	0.001
Sex				
Male	324 (32.9)	53 (22.0)	377 (30.7)	
Female	661 (67.1)	188 (78.0)	849 (69.2)	
Age (yr)	73.94±6.04	75.53±6.39	74.25±6.14	<0.001
60-69	234 (23.7)	38 (15.8)	272 (22.2)	0.022
70-79	558 (56.6)	146 (60.6)	704 (57.4)	
≥80	193 (19.6)	57 (23.6)	250 (20.4)	
Education				0.001
<Middle school graduation	645 (65.5)	186 (77.2)	831 (67.8)	
Middle school graduation to two-year college graduation	268 (27.2)	49 (20.3)	317 (25.8)	
≥Four-year university dropout	72 (7.3)	6 (2.4)	78 (6.4)	
Allowance (10^4^ KRW/mo)^3^	950	227	1,177	0.129
<30.00	678 (71.4)	177 (78.0)	855 (72.6)	
30.00-69.99	196 (20.6)	37 (16.0)	233 (19.8)	
≥70.00	76 (8.0)	13 (6.0)	89 (7.6)	
Smoking	48 (4.9)	13 (5.4)	61 (5.0)	0.739
Alcohol drinking	206 (20.9)	44 (18.3)	250 (20.4)	0.359
Household type				<0.001
Living alone	289 (29.3)	118 (49.0)	407 (33.2)	
Living with family	696 (70.7)	123 (51.0)	819 (66.8)	
Disease status (yes)^4^	456 (46.3)	146 (60.6)	602 (49.1)	<0.001
Physical activity (MET min/wk)	977	239	1,216	0.003
<600	591 (60.5)	169 (70.7)	760 (62.5)	
≥600	386 (39.5)	70 (29.3)	456 (37.5)	
Body mass index (kg/m^2^)	963	234	1,197	0.681
<23.0	409 (42.5)	93 (39.7)	502 (41.9)	
23.0-24.9	243 (25.2)	59 (25.2)	302 (25.2)	
≥25.0	311 (32.2)	82 (35.0)	393 (32.8)	
Total energy intake, n=385 (kcal/d)	1,804.80±352.37	1,470.37±911.29	1,707.51±881.94	0.001
Weekly intake (times/wk)				
Fruit				<0.001
Never	95 (9.6)	48 (19.9)	143 (11.7)	
<1	120 (12.2)	34 (14.1)	154 (12.6)	
1-3	281 (28.5)	81 (33.6)	362 (29.5)	
4-6	158 (16.0)	22 (9.1)	180 (14.7)	
Everyday	331 (33.6)	56 (23.2)	387 (31.6)	
Vegetable				0.228
Never	32 (3.2)	14 (5.8)	46 (3.7)	
<1	25 (2.5)	3 (1.2)	28 (2.3)	
1-3	107 (10.9)	29 (12.0)	136 (11.1)	
4-6	103 (10.4)	21 (8.7)	124 (10.1)	
Everyday	718 (72.9)	174 (72.2)	892 (72.7)	

Values are presented as number (%) or mean±standard deviation. SGDS-K, Korean version of the Short form Geriatric Depression Scale; KRW, Korean won; MET, metabolic equivalents of task.

1 SGDS-K≥8.

2 To derive p-values, t-test was used for continuous variables, and chi-square test was used for categorical variables.

3 10,000 KRW (roughly, US$10).

4 Admission/surgery/emergency room visit within 1 year, visit to a doctor without admission within 2 weeks.

**Table 2.** Cross-sectional multiple linear regression models for the association of weekly fruit/vegetable intake with SGDS-K and depression status (n=1,226)

Weekly intake		Crude model	p-value	Age- and sex-adjusted model	p-value	Multiple covariate-adjusted model^1^	p-value
Fruit (times/wk)							
	SGDS-K, β (95% CI)						
	Never	0.00		0.00		0.00	
	<1	-0.17 (-0.27, -0.07)	0.001	-0.17 (-0.28, -0.07)	<0.001	-0.17 (-0.28, -0.05)	0.005
	1-3	-0.26 (-0.35, -0.17)	<0.001	-0.24 (-0.33, -0.16)	<0.001	-0.17 (-0.27, -0.07)	0.001
	4-6	-0.61 (-0.72, -0.50)	<0.001	-0.55 (-0.66, -0.44)	<0.001	-0.42 (-0.54, -0.29)	<0.001
	Everyday	-0.53 (-0.62, -0.44)	<0.001	-0.50 (-0.59, -0.41)	<0.001	-0.33 (-0.44, -0.21)	<0.001
	Depression status, OR (95% CI)						
	Never	1.00 (reference)		1.00 (reference)		1.00 (reference)	
	<1	0.56 (0.33, 0.94)	0.028	0.55 (0.33, 0.93)	0.025	0.65 (0.39, 1.07)	0.087
	1-3	0.57 (0.37, 0.87)	0.010	0.58 (0.38, 0.90)	0.014	0.77 (0.51, 1.16)	0.205
	4-6	0.28 (0.16, 0.48)	<0.001	0.30 (0.17, 0.53)	<0.001	0.42 (0.23, 0.75)	0.003
	Everyday	0.33 (0.21, 0.52)	<0.001	0.36 (0.23, 0.57)	<0.001	0.55 (0.34, 0.89)	0.015
Vegetable (times/wk)							
	SGDS-K, β (95% CI)						
	Never	0.00		0.00		0.00	
	<1	-0.68 (-0.95, -0.43)	<0.001	-0.70 (-0.96, -0.44)	<0.001	-0.86 (-1.18, -0.55)	<0.001
	1-3	-0.20 (-0.35, -0.05)	0.010	-0.14 (-0.29, 0.01)	0.062	-0.18 (-0.35, -0.01)	0.038
	4-6	-0.53 (-0.69, -0.36)	<0.001	-0.43 (-0.59, -0.27)	<0.001	-0.36 (-0.53, -0.18)	<0.001
	Everyday	-0.30 (-0.43, -0.17)	<0.001	-0.22 (-0.35, -0.08)	0.001	-0.15 (-0.29, 0.00)	0.048
	Depression status, OR (95% CI)						
	Never	1.00 (reference)		1.00 (reference)		1.00 (reference)	
	<1	0.27 (0.07, 1.06)	0.061	0.26 (0.07, 1.02)	0.054	0.22 (0.04, 1.10)	0.065
	1-3	0.62 (0.29, 1.31)	0.211	0.69 (0.32, 1.48)	0.339	0.62 (0.26, 1.47)	0.282
	4-6	0.47 (0.21, 1.02)	0.056	0.55 (0.25, 1.23)	0.147	0.59 (0.25, 1.43)	0.245
	Everyday	0.55 (0.29, 1.06)	0.075	0.65 (0.34, 1.26)	0.204	0.70 (0.33, 1.48)	0.353

SGDS-K, Korean version of the Short form Geriatric Depression Scale; OR, odds ratio; CI, confidence interval; KRW, Korean won; MET, metabolic equivalents of task.

1 Adjusted for age, sex, education, allowance (10,000 KRW/mo), smoking status, alcohol consumption status, household type, disease status, physical activity (MET min/wk), and body mass index.

**Table 3.** Generalized linear mixed-effects models for the association of weekly fruit/vegetable intake with SGDS-K and depression status (n=305; 915 observations)

Weekly intake		Crude model	p-value	Age- and sex-adjusted model	p-value	Multiple covariate-adjusted model^1^	p-value
Fruit (times/wk)							
	SGDS-K, β (95% CI)						
	Never	0.00		0.00		0.00	
	<1	0.05 (-0.12, 0.22)	0.566	0.05 (-0.11, 0.22)	0.547	0.08 (-0.09, 0.26)	0.353
	1-3	-0.09 (-0.24, 0.06)	0.218	-0.09 (-0.24, 0.05)	0.204	-0.09 (-0.24, 0.07)	0.205
	4-6	-0.14 (-0.32, 0.04)	0.132	-0.14 (-0.32, 0.05)	0.141	-0.13 (-0.32, 0.06)	0.167
	Everyday	-0.12 (-0.29, 0.05)	0.167	-0.11 (-0.28, 0.05)	0.187	-0.10 (-0.28, 0.07)	0.244
	Depression status, OR (95% CI)						
	Never	1.00 (reference)		1.00 (reference)		1.00 (reference)	
	<1	1.33 (0.47, 3.72)	0.591	1.41 (0.51, 3.92)	0.511	1.34 (0.48, 3.71)	0.574
	1-3	0.43 (0.17, 1.08)	0.071	0.45 (0.18, 1.13)	0.089	0.66 (0.27, 1.60)	0.356
	4-6	0.32 (0.10, 0.95)	0.041	0.36 (0.12, 1.08)	0.068	0.53 (0.18, 1.56)	0.248
	Everyday	0.21 (0.08, 0.53)	0.001	0.25 (0.10, 0.65)	0.004	0.40 (0.16, 1.02)	0.056
Vegetables (times/wk)							
	SGDS-K, β (95% CI)						
	Never	0.00		0.00		0.00	
	<1	0.18 (-0.22, 0.59)	0.369	0.17 (-0.22, 0.57)	0.396	0.11 (-0.31, 0.52)	0.613
	1-3	0.10 (-0.25, 0.47)	0.569	0.10 (-0.25, 0.45)	0.566	-0.03 (-0.40, 0.33)	0.856
	4-6	-0.02 (-0.38, 0.36)	0.922	-0.01 (-0.38, 0.35)	0.942	-0.18 (-0.56, 0.20)	0.357
	Everyday	0.11 (-0.22, 0.46)	0.512	0.12 (-0.22, 0.45)	0.498	0.06 (-0.29, 0.41)	0.745
	Depression status, OR (95% CI)						
	Never	1.00 (reference)		1.00 (reference)		1.00 (reference)	
	<1	3.71 (0.30, 46.42)	0.308	3.43 (0.29, 40.96)	0.331	1.76 (0.16, 19.16)	0.644
	1-3	1.90 (0.20, 17.66)	0.572	1.97 (0.22, 17.50)	0.544	0.96 (0.12, 7.91)	0.968
	4-6	1.18 (0.12, 11.87)	0.885	1.29 (0.13, 12.41)	0.824	0.79 (0.09, 7.21)	0.832
	Everyday	1.83 (0.22, 14.98)	0.572	2.01 (0.26, 15.84)	0.506	1.58 (0.22, 11.29)	0.648

SGDS-K, Korean version of the Short form Geriatric Depression Scale; OR, odds ratio; CI, confidence interval; KRW, Korean won; MET, metabolic equivalents of task.

1 Adjusted for age, sex, education, allowance (10,000 KRW/mo), smoking status, alcohol consumption status, household type, disease status, physical activity (MET min/wk), and body mass index.

**Table 4.** The dose-dependent relationship between the weekly fruit/vegetable intake and SGDS-K (β) and depression status (OR)^1^

Variables	β (95% CI)	p-value	OR (95% CI)	p-value
Cross-sectional multiple linear regression model (n=1,226)				
Weekly fruit intake	-0.04 (-0.06, -0.03)	<0.001	0.90 (0.84, 0.97)	0.004
Weekly vegetable intake	0.01 (-0.00, 0.03)	0.151	1.02 (0.94, 1.10)	0.672
Generalized linear mixed-effects model (n=305; 915 observations)				
Weekly fruit intake	-0.04 (-0.06, -0.03)	<0.001	0.87 (0.78, 0.96)	0.006
Weekly vegetable intake	0.00 (-0.01, 0.02)	0.631	1.07 (0.94, 1.22)	0.330

SGDS-K, Korean version of the Short form Geriatric Depression Scale; OR, odds ratio; CI, confidence interval; KRW, Korean won; MET, metabolic equivalents of task.

1 Adjusted for age, sex, education, allowance (10,000 KRW/mo), smoking status, alcohol consumption status, household type, disease status, physical activity (MET min/wk), and body mass index.

## SUPPLEMENTARY MATERIALS

Supplementary Material 1. Follow-up rate of the KEEP-II participants from 2012 to 2014 in Seoul and Asan

Supplementary Material 2.

Supplementary Material 3. Further adjustment with weekly vegetable(fruit) intake for weekly fruit(vegetable) intake in multiple covariate-adjusted models

Supplementary Material 4. The dose-dependent relationship between the weekly fruit/vegetable intake and SGDS-K (β) and depression status (OR) regarding Supplementary Material 3.

Supplementary Material 5. Multiple covariate-adjusted models* after combining "never" and "less than 1 time" in weekly fruit/vegetable

Supplementary Material 6. The dose-dependent relationship between the weekly fruit/vegetable intake and SGDS-K (β) and depression status (OR) regarding Supplementary Material 5.
