# Supplementary material for: Association between weekly fruit and vegetable consumption and depressive symptoms: results from the Korean Elderly Environmental Panel study
Source: Epidemiol Health. 2021 Apr 20;43:e2021029. doi: 10.4178/epih.e2021029 (PMC8289474; doi:10.4178/epih.e2021029)
Supplement: Supplementary Material 1. — Follow-up rate of the KEEP-II participants from 2012 to 2014 in Seoul and Asan [file epih-43-e2021029-suppl1.docx]

**Supplementary Material 1**. Follow-up rate of the KEEP-II participants from 2012 to 2014 in Seoul and Asan


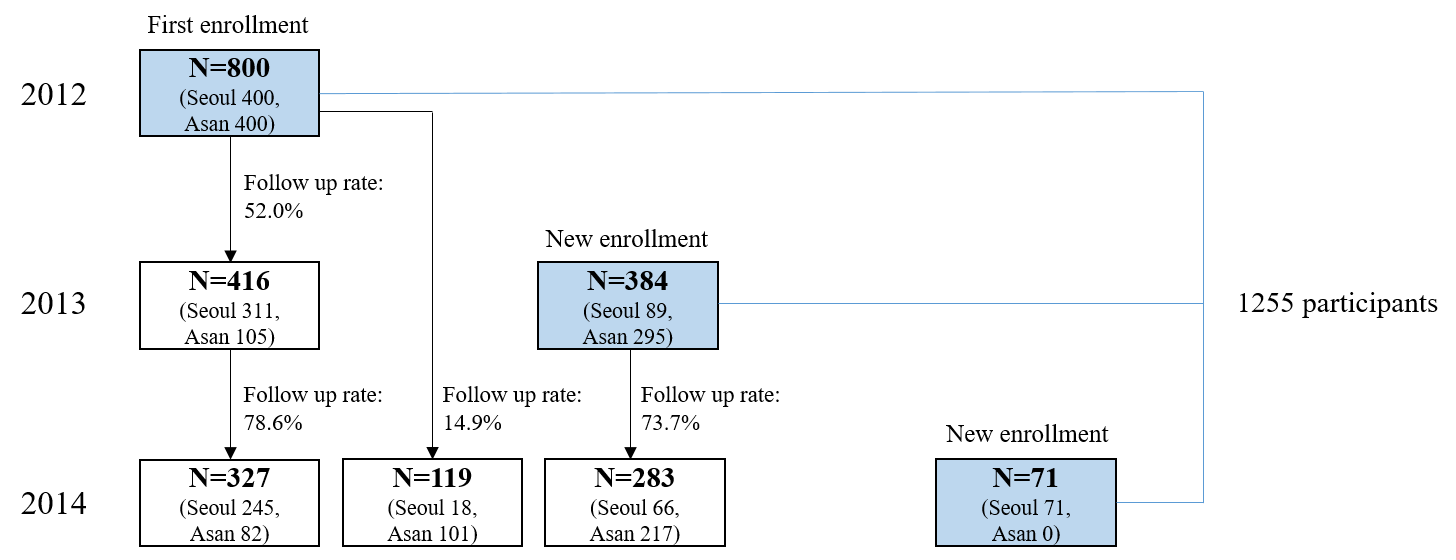


**Supplementary Material 2.**

Definition of covariates

1) Education level

(a) <middle school graduation, including “never been to school,” “elementary school dropout,” and “elementary school graduation/currently on middle school/middle school dropout”; (b) middle school graduation to two-year college, including “middle school graduation/currently on high school/high school dropout,” “high school graduation,” and “two-year college graduation”; (c) ≥four-year university dropout, including “four-year university dropout,” “currently on four-year university/graduation,” and “postgraduate and above.”

2) Physical activities

The frequency and duration of light, moderate, and severe physical activities were coded as 1.5 MET minutes, 4.5 MET minutes, and 8 MET minutes, respectively. The product of the frequency, duration (min), and intensity of exercise performed per week provided the total MET minutes per week.

| **Supplementary Material 3**. Further adjustment with weekly vegetable(fruit) intake for weekly fruit(vegetable) intake in multiple covariate-adjusted models^*^ | | | | | | | | |
| --- | --- | --- | --- | --- | --- | --- | --- | --- |
| Cross-sectional multiple linear regression models (N=1,226) | | | | | | | | |
| Weekly fruit intake | | β | (95% CI) | p-value |  | OR | (95% CI) | p-value |
|  | Never | 0 |  |  |  | 1 |  |  |
|  | less than 1 time | –0.11 | (–0.23, 0.01) | 0.074 |  | 0.57 | (0.31, 1.07) | 0.080 |
|  | 1-3 times | –0.17 | (–0.27, –0.06) | 0.002 |  | 0.69 | (0.41, 1.16) | 0.156 |
|  | 4-6 times | –0.37 | (–0.51, –0.24) | <0.001 |  | 0.32 | (0.16, 0.64) | 0.001 |
|  | Everyday | –0.35 | (–0.46, –0.23) | <0.001 |  | 0.43 | (0.24, 0.77) | 0.005 |
| Weekly vegetable intake | | β | (95% CI) | p-value |  | OR | (95% CI) | p-value |
|  | Never | 0 |  |  |  | 1 |  |  |
|  | less than 1 time | –0.81 | (–1.14, –0.50) | <0.001 |  | 0.28 | (0.05, 1.47) | 0.133 |
|  | 1-3 times | –0.12 | (–0.29, 0.06) | 0.197 |  | 0.72 | (0.29, 1.76) | 0.469 |
|  | 4-6 times | –0.24 | (–0.43, –0.05) | 0.011 |  | 0.86 | (0.34, 2.17) | 0.752 |
|  | Everyday | –0.05 | (–0.20, 0.11) | 0.550 |  | 0.93 | (0.42, 2.05) | 0.860 |
| Generalized linear mixed-effect models (N=305 (915 observations)) | | | | | | | | |
| Weekly fruit intake | | β | (95% CI) | p-value |  | OR | (95% CI) | p-value |
|  | Never | 0 |  |  |  | 1 |  |  |
|  | less than 1 time | 0.09 | (–0.09, 0.26) | 0.333 |  | 1.15 | (0.41, 3.27) | 0.792 |
|  | 1-3 times | –0.08 | (–0.24, 0.07) | 0.283 |  | 0.51 | (0.20, 1.27) | 0.148 |
|  | 4-6 times | –0.13 | (–0.32, 0.06) | 0.185 |  | 0.45 | (0.15, 1.35) | 0.153 |
|  | Everyday | –0.11 | (–0.29, 0.06) | 0.201 |  | 0.31 | (0.12, 0.82) | 0.018 |
| Weekly vegetable intake | | β | (95% CI) | p-value |  | OR | (95% CI) | p-value |
|  | Never | 0 |  |  |  | 1 |  |  |
|  | less than 1 time | 0.09 | (–0.33, 0.51) | 0.668 |  | 1.87 | (0.16, 21.84) | 0.617 |
|  | 1-3 times | –0.01 | (–0.38, 0.36) | 0.974 |  | 1.30 | (0.15, 11.64) | 0.812 |
|  | 4-6 times | –0.18 | (–0.56, 0.21) | 0.365 |  | 0.90 | (0.09, 8.85) | 0.928 |
|  | Everyday | 0.07 | (–0.27, 0.42) | 0.679 |  | 2.15 | (0.28, 16.68) | 0.463 |

^*^ Adjusted for age, sex, education, allowance (10,000KRW/month), smoking status, alcohol consumption status, household type, disease status, physical activity (MET minutes per week) and body mass index

| **Supplementary Material 4.** The dose-dependent relationship between the weekly fruit/vegetable intake and SGDS-K (β) and depression status (OR) regarding Supplementary Material 3. | | | | | |
| --- | --- | --- | --- | --- | --- |
| Cross-sectional multiple linear regression model (multiple covariate-adjusted) (N=1,226) | | | | | |
|  |  | β (95% CI) | p-value | OR (95% CI) | p-value |
| Weekly fruit intake | | –0.05 (–0.06, –0.03) | <0.001 | 0.90 (0.84, 0.96) | 0.000 |
| Weekly vegetable intake | | 0.02 (0.00, 0.04) | 0.011 | 1.04 (0.96, 1.12) | 0.337 |
| Generalized linear mixed-effect model (multiple covariate-adjusted) (N=305 (915 observations)) | | | | | |
|  |  | β (95% CI) | p-value | OR (95% CI) | p-value |
| Weekly fruit intake | | –0.05 (–0.06, 0.03) | <0.001 | 0.84 (0.75, 0.93) | 0.001 |
| Weekly vegetable intake | | 0.01 (–0.00, 0.03) | 0.149 | 1.10 (0.96, 1.26) | 0.153 |

^*^ Covariates used for multiple covariate-adjusted model: age, sex, education, allowance (10,000KRW/month), smoking status, alcohol consumption status, household type, disease status, physical activity (MET minutes per week) and body mass index

^†^ “never” is included in “less than 1 time”

| **Supplementary Material 5.** Multiple covariate-adjusted models^*^ after combining "never" and "less than 1 time" in weekly fruit/vegetable intake | | | | | | | | |
| --- | --- | --- | --- | --- | --- | --- | --- | --- |
| Cross-sectional multiple linear regression models (N=1,226) | | | | | | | | |
| Weekly fruit intake | | β | (95% CI) | p-value |  | OR | (95% CI) | p-value |
|  | less than 1 time^†^ | 0 |  |  |  | 1 |  |  |
|  | 1-3 times | –0.08 | (–0.16, 0.00) | 0.056 |  | 0.92 | (0.61, 1.40) | 0.707 |
|  | 4-6 times | –0.33 | (–0.44, –0.22) | <0.001 |  | 0.44 | (0.24, 0.79) | 0.007 |
|  | Everyday | –0.24 | (–0.34, –0.14) | <0.001 |  | 0.60 | (0.37, 0.98) | 0.043 |
| Weekly vegetable intake | | β | (95% CI) | p-value |  | OR | (95% CI) | p-value |
|  | less than 1 time | 0 |  |  |  | 1 |  |  |
|  | 1-3 times | 0.06 | (–0.09, 0.22) | 0.427 |  | 0.96 | (0.44, 2.11) | 0.927 |
|  | 4-6 times | –0.11 | (–0.28, 0.05) | 0.181 |  | 0.91 | (0.41, 2.05) | 0.829 |
|  | Everyday | 0.09 | (–0.04, 0.23) | 0.177 |  | 1.08 | (0.56, 2.09) | 0.818 |
| Generalized linear mixed-effects models (N=305 (915 observations)) | | | | | | | | |
| Weekly fruit intake | | β | (95% CI) | p-value |  | OR | (95% CI) | p-value |
|  | less than 1 time | 0 |  |  |  | 1 |  |  |
|  | 1-3 times | –0.13 | (–0.25, –0.01) | 0.035 |  | 0.47 | (0.22, 0.99) | 0.046 |
|  | 4-6 times | –0.18 | (–0.34, –0.01) | 0.034 |  | 0.42 | (0.16, 1.08) | 0.072 |
|  | Everyday | –0.15 | (–0.30, 0.00) | 0.051 |  | 0.32 | (0.15, 0.71) | 0.005 |
| Weekly vegetable intake | | β | (95% CI) | p-value |  | OR | (95% CI) | p-value |
|  | less than 1 time | 0 |  |  |  | 1 |  |  |
|  | 1-3 times | –0.11 | (–0.33, 0.12) | 0.365 |  | 0.76 | (0.19, 3.09) | 0.703 |
|  | 4-6 times | –0.25 | (–0.51, 0.01) | 0.060 |  | 0.66 | (0.13, 3.22) | 0.605 |
|  | Everyday | –0.01 | (–0.21, 0.19) | 0.904 |  | 1.26 | (0.38, 4.23) | 0.705 |

| **Supplementary Material 6.** The dose-dependent relationship between the weekly fruit/vegetable intake and SGDS-K (β) and depression status (OR) regarding Supplementary Material 5. | | | | | |
| --- | --- | --- | --- | --- | --- |
| Cross-sectional multiple linear regression model (multiple covariate-adjusted) (N=1226) | | | | | |
|  |  | β (95% CI) | p-value | OR (95% CI) | p-value |
| Weekly fruit intake | | –0.25 (–0.36, –0.14) | <0.001 | 0.44 (0.24, 0.81) | 0.008 |
| Weekly vegetable intake | | –0.18 (–0.32, –0.03) | 0.016 | 0.90 (0.43, 1.85) | 0.766 |
| Generalized linear mixed-effect model (multiple covariate-adjusted) (N=305 (915 observations)) | | | | | |
|  |  | β (95% CI) | p-value | OR (95% CI) | p-value |
| Weekly fruit intake | | –0.04 (–0.06, –0.03) | <0.001 | 0.86 (0.78, 0.96) | 0.005 |
| Weekly vegetable intake | | 0.01 (–0.01, 0.02) | 0.559 | 1.07 (0.94, 1.22) | 0.281 |
